# Correlation between religiosity, spirituality and quality of life in adolescents with and without cleft lip and palate*

**DOI:** 10.1590/1518-8345.2498-3059

**Published:** 2018-10-25

**Authors:** Francely Tineli Farinha, Fábio Luiz Banhara, Gesiane Cristina Bom, Lilia Maria Von Kostrisch, Priscila Capelato Prado, Armando dos Santos Trettene

**Affiliations:** 1 Universidade de São Paulo, Hospital de Reabilitação de Anomalias Craniofaciais, Bauru, SP, Brazil.; 2 Prefeitura Municipal de Fortaleza, Secretaria da Saúde, Fortaleza, CE, Brazil.

**Keywords:** Spirituality, Religion, Quality of Life, Adolescent, Cleft Lip, Cleft Palate

## Abstract

**Objective:**

to correlate spirituality and religiosity with quality of life of adolescents with and without cleft lip and palate.

**Methods:**

cross-sectional and correlational study involving two groups: case group (n = 40) and comparison group (n = 40). The Duke University Religion Index (DUREL) and the World Health Organization Quality of Life Bref were used for data collection. The Mann-Whitney, Chi-Square, Student’s t-test and Pearson’s correlation tests were used in the statistical analyses, with a significance level of 5% (p ≤ 0.05).

**Results:**

organizational religiosity and overall quality of life were significantly higher in the case group (p = 0.031 and p = 0.012, respectively). As for quality of life, the Environment Domain was significantly higher in the case group (p < 0.001). In the correlation between religiosity and spirituality, non-organizational religiosity had a strong correlation (r = 0.62) with organizational religiosity (p < 0.001). In the correlation of religiosity and spirituality with quality of life, only a moderate correlation between spirituality and overall quality of life was identified (r = -0.35, p = 0.026).

**Conclusion:**

there was no relationship of religiosity and spirituality with quality of life among adolescents with cleft lip and palate for most aspects evaluated.

## Introduction

Individuals with cleft lip and palate may present functional, aesthetic and psychosocial problems. Initially, functional problems are prevalent, particularly in feeding. During childhood and adolescence, however, aesthetic and especially psychosocial problems stand out^1^ ^-^ ^2^.

Adolescence is a phase characterized by biopsychosocial transformations, socialization and exacerbation of aesthetic standards imposed by modernist and globalized society. Adolescents with cleft lip and palate may face discrimination and prejudice, generating stigmatization, which have a clear influence on their social life and, consequently, quality of life^3^.

These adolescents may experience moments of denial, intellectualization, depression and exaggerated behaviors. However, these reactions are directly related to the establishment of social, family and cultural relations and, consequently, influence their self-esteem and quality of life. Dissatisfaction promotes in individuals feelings of inferiority, weakness, rejection and impotence that can lead to failures in the rehabilitation process^4^.

In the process of coping, there are factors that act as additional resources to treatment and rehabilitation, among which are spirituality and religiosity^5^.

Spirituality refers to the awareness that there is something sacred, and involves particular values and concepts of each individual, while religiosity relates to activities developed collectively, encompassing a system of defined or pre-established beliefs, dogmas and practices^6^.

The benefits of spirituality and/or religiosity among adolescents include lower depression and anxiety, more happiness and satisfaction with life, better perception of quality of life, reduction of stress, and protection from behaviors that pose risk to health such as sexual activities and use of licit and illicit drugs^7^ ^-^ ^12^.

A growing number of studies has evaluated religiosity and spirituality, as well as their impact on people’s quality of life^6^. Quality of life is an indicator of health and its evaluation in distinct populations, including adolescents, is fundamental. Adolescence is known to be one of the most important phases in the formation of a population and low levels of health in this phase can affect health during adulthood^7^.

Considering the vulnerability of adolescents with cleft lip and palate to aesthetic and psychosocial problems, as well as the evidence of the benefits of spirituality and religiosity on the modalities of situational coping, it is relevant to understand their influence on the quality of life of this population.

Our hypothesis was that adolescents with cleft lip and palate presented higher levels of spirituality and religiosity, and this had a consequent positive influence on their perception of quality of life compared to adolescents without cleft lip and palate.

No studies were found with this approach in the databases consulted and this emphasizes the importance and novelty of this theme. The objective of this investigation was to correlate spirituality and religiosity with quality of life of adolescents with and without cleft lip and palate.

## Methods

This is an exploratory, cross-sectional, correlational study with quantitative approach.

Comparisons were made between two groups: a case group and a comparison group. The population in the case group consisted of adolescents who were hospitalized for secondary surgical procedures, including rhinoplasty, septoplasty and alveolar bone graft. Inclusion criteria were age between 15 and 18 years, and presence operated unilateral cleft lip and palate, and absence of associated syndromes or neurological diseases.

The comparison group consisted of adolescents without cleft lip and palate who were students of a public school. In this group, the inclusion criterion was to be aged between 15 and 18 years and to join the research.

Adolescents who reported using psychoactive drugs such as anxiolytics, hypnotics, antidepressants, antipsychotics and mood stabilizers, as well as illicit drugs, were excluded from the study groups.

Sampling was consecutive and non-probabilistic. The following correlation coefficients were adopted to interpret the magnitude of the correlations: < 0.4 (low magnitude), ≥ 0.4 to < 0.5 (moderate magnitude) and ≥ 0.5 (strong magnitude)^13^. A moderate correlation of 0.45^13^, a power of 80% and a significance level of 5% was considered for the sample calculation; the minimum sample estimated was of 37 participants per group. Finally, 40 participants were chosen to compose the sample in each group (case and comparison).

The participants were initially invited to join the study, the objectives were explained and the instruments of data collection were presented. Three instruments were used to collect data: a Sociodemographic Questionnaire, the Duke University Religion Index (DUREL)^14^ to assess religiosity and spirituality, and the World Health Organization Quality of Life (WHOQOL-Bref) to assess quality of life.

The Sociodemographic Questionnaire was used to characterize the participants according to the variables: age, sex, schooling, religion and socioeconomic classification^16^, number of children, occupation and marital or affective status.

The information to verify the inclusion and exclusion criteria in the case group was obtained by consulting medical records, as well as information in the sociodemographic characterization. In the comparison group, the inclusion and exclusion criteria as well as sociodemographic information were obtained through individual interviews in a private setting.

Data were collected from a group of participants in the preoperative period, individually, because the presence of edema, pain, discomfort and functional limitations may interfere with the individual’s responses and perception. As for the comparative group, the educational institution provided an auditorium for the interviews, which lasted 30 minutes, on average.

The DUREL Scale^14^ and the WHOQOL-Bref^15^ are self-applicable instruments. They were given to the participants at the same time. Data were collected between August and November 2016.

The perception of quality of life of adolescents was evaluated through the validated Portuguese version of the WHOQOL-Bref^15^. This instrument can be used to evaluate the quality of life of both healthy populations and people affected by chronic diseases^17^. Although this instrument has not been validated for adolescents in Brazil, a research showed that this instrument has valid content and adequate psychometric properties to measure the quality of life of adolescents^18^. In addition, in the present study, the Cronbach’s alpha value for the WHOQOL-Bref application was 0.84, indicating good internal consistency.

The WHOQOL-Bref contains 26 questions, among which 24 are distributed in four domains: Physical, Psychological, Social Relations and Environment. The other two questions address the perception of overall quality of life and satisfaction with one’s own health. Each domain has a score ranging from zero to 100 where zero corresponds to worse quality of life and 100 to better quality of life^19^.

The DUREL Scale was concomitantly applied. This scale consists of five items divided into three domains: organizational religiosity (OR), non-organizational religiosity (NOR) and intrinsic religiosity or spirituality (IR)^14^. In this study, IR was referred to as spirituality.

Organizational religiosity refers to attendance to religious meetings, such as services, masses, prayer groups, and so forth. The score ranges from one to six. Non-organizational religiosity is a trait that does not depend on other people, but rather refers to personal religious activity. It includes prayer, meditation, among others. It has a score ranging from one to six. Spirituality refers to the internalization and full experience of religiosity as the main objective, where individuals seek harmony with religious principles^14^.

The DUREL scale has a score that varies from three to fifteen. With regard to the calculation of this score, it is recommended that the three domains be analyzed separately. In each domain, the lower the score, the greater is the religiosity^14^.

The DUREL scale was translated and validated for the Brazilian population. Although not validated specifically for adolescents, its use in populations with diverse sociodemographic characteristics is encouraged^14^ ^,^ ^20^. In the present study, the Cronbach’s alpha value for the DUREL scale was 0.82, indicating good internal consistency.

The research received a favorable Opinion from the Committee of Ethics in Research Involving Human Subjects of the Hospital, through letter 1.614.101 and CAAE: 55837916.9.0000.5441. All the participants formalized their adherence by signing of the Informed Consent Form. Adolescents under 18 years of age formalized their adherence by signing the Informed Consent Form, so as their legal representatives also did, in accordance with Resolution 466/12 of the National Health Council.

Data were analyzed using the Statistical Package for Social Sciences (SPSS) software version 21.0 for Windows. The Chi-Square and Student t-tests were used to analyze of socio-demographic variables, sex and age in both groups. In order to compare the case and comparative groups in terms of religiosity, spirituality and quality of life, the Mann-Whitney test was used. Pearson’s correlation test was used in the case group to correlate the measures of interest, quality of life, religiosity and spirituality. An analysis of linear correlation strength between the measurements was also used. In this analysis, correlation values ​​smaller than 0.30 indicate a weak correlation despite statistical significance, which mean a correlation that is not clinically relevant; values ​​between 0.30-0.50 indicate a moderate correlation and above 0.50, strong correlation^21^. The significance level adopted for all tests was 5% (p ≤ 0.05).

## Results

Regarding the characterization of the case group, the mean age was 16.48 (± 1.04) years. As for sex, similarity was observed (50%; n = 20). In the other variables, secondary education (87.5%, n=35), single marital status (90.0%, n = 36) and evangelicals (47.5%, n = 19) prevailed.

As for the comparison group, the mean age was 16.38 (± 1.17) years, and there was a prevalence of female participants (60.0%, n=24), with secondary education (n=40, 100%), single (77.5%, n=31) and evangelicals (62.5%; n=25).

No statistically significant differences were found between the sociodemographic characteristics of the group participants, demonstrating homogeneity between them.

The case group was composed by adolescents with unilateral cleft lip and palate. Regarding the surgical procedure they were to perform, alveolar bone graft and rhinoseptoplasty had similar representation (both 50.0%, n = 20). As for origin, the Southeast region prevailed (55.0%, n=22).

In the analysis of spirituality and religiosity, we observed that the case group had higher median values compared to the comparative group in terms of organizational religiosity (p = 0.031) (Table 1).

**Table 1:** Analysis of religiosity and spirituality in the case group and comparative group. Bauru, SP, Brazil, 2016

**Variables**	**Groups**	**n**	**Median**	Q1*	**Q3** ^**†**^	**Mean**	**Standard deviation**	**p value**
**NOR** ^**‡**^	**Case**	**40**	**2.0**	**1.0**	**3.8**	**2.5**	**1.5**	**0.483**
	**Comparison**	**40**	**2.0**	**1.3**	**4.0**	**2.8**	**1.7**	
**OR** ^**§**^	**Case**	**40**	**3.0**	**2.0**	**5.0**	**3.5**	**1.7**	**0.031** ^**||**^
	**Comparison**	**40**	**4.5**	**3.0**	**6.0**	**4.3**	**1.6**	
**Spirituality**	**Case**	**40**	**5.0**	**4.0**	**6.0**	**5.7**	**2.4**	**0.171**
	**Comparison**	**40**	**6.0**	**4.0**	**8.8**	**6.7**	**3.2**	

*Q1=1^st^Quartile; †Q3=3^rd^Quartile; ‡NOR= Non-organizational religiosity; §OR= Organizational religiosity; ||Mann-Whitney test with significance level adopted of 5% (p ≤ 0.05).

Regarding quality of life, it was observed that the case group had higher median values than the comparative group, in relation to the overall quality of life (p = 0.012). As for the domains, it was observed that the case group presented higher median values, compared to the comparative group in the Environment domain (p < 0.001) (Table 2).

**Table 2:** Analysis of Quality of Life in the Case Group and Comparative Group. Bauru, SP, Brazil, 2016

Domains	Groups	N	Median	Q1*	Q3^†^	Mean	Standard deviation	p value
Physical	Case	40	81.0	69.0	88.0	80.6	9.6	0.141
	Comparison	40	81.0	63.0	86.3	75.6	14.0	
Psychological	Case	40	75.0	69.0	81.0	74.2	13.3	0.657
	Comparison	40	75.0	69.0	81.0	72.6	13.6	
Social relationships	Case	40	75.0	69.0	81.0	77.7	13.8	0.056
	Comparison	40	69.0	51.5	81.0	68.0	20.8	
Environment	Case	40	81.0	69.0	86.3	75.5	12.2	<0.001^‡^
	Comparison	40	63.0	50.0	73.5	61.7	14.3	
Overall quality of life	Case	40	4.0	4.0	5.0	4.4	0.7	0.012^‡^
	Comparison	40	4.0	4.0	4.0	4.0	0.7	
General perception of health	Case	40	4.0	4.0	5.0	4.0	1.1	0.818
	Comparison	40	4.0	3.3	5.0	4.0	1.1	

*Q1=1^st^ Quartile; †Q3= 3^rd^ Quartile; ‡Mann-Whitney test with significance level of 5% (p ≤ 0.05).

As for the correlation between religiosity and spirituality, NOR presented a strong correlation (r = 0.62) with OR (p < 0.001), while spirituality presented a moderate correlation with NOR (r = 0.44) and with OR (r = 0.43) (p = 0.005 and p = 0.006, respectively) (Table 3).

**Table 3:** Distribution of the correlation of religiosity and spirituality applied in the Case Group. Bauru, SP, Brazil, 2016

Correlation variables	r*	Correlation^†^	p value^‡^
NOR^§^/OR^||^	0.62	Strong	<0.001
NOR^||^/Spirituality	0.44	Moderate	0.005
OR^§^/Spirituality	0.43	Moderate	0.006

*Pearson correlation; †Linear correlation; ‡Level of significance adopted of 5% (p ≤ 0.05); §NOR = Non-organizational religiosity; ||OR = Organizational religiosity.

The analysis of correlation between domains of quality of life with religiosity and spirituality, a moderate correlation (r = -0.35) between spirituality and overall quality of life (p = 0.026) was identified; only one of the correlations was significant, indicating that there is no relationship between these variables in most of the aspects evaluated. It is noteworthy that the values in the spirituality scale are inversely proportional, justifying the negative value of the correlations (Table 4).

**Table 4:** Correlation between quality of life, religiosity and spirituality in the Case Group. Bauru, SP, Brazil, 2016

Correlation variables		r*	Correlation^†^	p value
Domains/NOR^‡^	Physical	0.21	Weak	0.193
	Psychological	-0.01	Weak	0.942
	Social relationships	-0.08	Weak	0.626
	Environment	0.19	Weak	0.234
	Overall quality of life	0.04	Weak	0.806
	General perception of health	0.07	Weak	0.649
Domains/OR^§^	Physical	0.03	Weak	0.848
	Psychological	-0.14	Weak	0.404
	Social relationships	-0.16	Weak	0.336
	Environment	-0.14	Weak	0.390
	Overall quality of life	0.07	Weak	0.670
	General perception of health	0.25	Weak	0.117
Domains/ Spirituality	Physical	-0.07	Weak	0.663
	Psychological	-0.23	Weak	0.162
	Social relationships	-0.26	Weak	0.101
	Environment	0.08	Weak	0.619
	Overall quality of life	-0.35	Moderate	0.026^||^
	General perception of health	-0.17	Weak	0.286

*Pearson correlation; †Analysis of linear correlation forces; ‡NOR= Non-organizational religiosity; §OR = Organizational religiosity; ||Level of significance adopted of 5% (p ≤ 0.05).

## Discussion

The mean age of participants was 16.48 years. It is in this age group that the aesthetics and social interactions are in evidence and are of extreme importance for the development of individuals^22^.

Adolescents with cleft lip and palate not only deal with problems of daily life and age but also find themselves surrounded by concerns with the malformation, including concern with scars and quality of voice. These limitations lead to problems of discrimination and stigmatization in society, what certainly influences social relations and the perception of quality of life.

This relationship regarding the affective characterization of the participants was observed in the present study, where only 10% of the adolescents with fissure reported being dating, against 22.5% of the adolescents without fissure.

Regarding socioeconomic classification, the lower class prevailed. Individuals belonging to the most favored classes are concerned with cognitive, cultural and beauty issues in equal proportion, while the less favored classes worry more with intelligence^23^.

Research indicated that the level of self-esteem in adolescents with cleft lip and palate was significantly lower than peers without this malformation, especially in the female gender. These adolescents carry with them their physical defects or scars and all that these traits can mean in social sense, embracing them as part of their identity. However, what this stigma can mean and cause depends on several factors, including subjective and reflexive issues leading to complex psychological problems^4^.

Regarding the origin, adolescents from the Southeast region prevailed in the case group. This result is associated to the fact that the Institution is located in this region. It should be emphasized that the process of rehabilitation should be guided by interdisciplinary care and carried out in centers of excellence.

There was a equivalence of sexes among the participants of the case group. The literature indicates a predominance of unilateral cleft lip and palate in the male sex, while isolated palate clefts are prevalent in females^24^.

Regarding the classification of clefts, unilateral and bilateral cleft lip and palate is the one that promotes greater functional and aesthetic impairment because of the anatomical area involved. In this context, the rehabilitation protocol is extensive and complex. The results of surgeries in early childhood can directly affect the self-esteem of these individuals when they become adolescents and, consequently, their interaction and acceptance in society^1^ ^-^ ^3^.

In this investigation, religiosity was evaluated in both groups, and the vast majority of adolescents reported having a religion, predominantly evangelical. According to the Brazilian Institute of Geography and Statistics (IBGE), approximately 86% of the Brazilian population is self-declared Christian, with prevalence of the catholic religion. According to the IBGE, the proportion of Catholics was higher among people over 40 years of age, while Pentecostal evangelicals predominated among children and adolescents, according to what was found in this study^25^.

The influence of spirituality and religiosity as a form of coping with different pathologies and unfavorable contexts is explored in the literature^26^ ^-^ ^28^. Investigations comparing healthy populations and individuals with diseases identified that people with health problems presented better spirituality and religiosity scores^27^ ^-^ ^28^.

In the present study, adolescents with cleft lip and palate presented high organizational religiosity in comparison to adolescents without cleft. They attended more often churches or temples and had greater social interaction in the religious community. We also observed a correlation between organizational and non-organizational religiosity and spirituality in the group of adolescents with cleft lip and palate. This finding confirms that both are intrinsically intertwined and, therefore, represent a construction^28^.

The correlation between spirituality and religiosity acts as a protection factor for maintenance of health and well-being among adolescents. It is an important joint construction in the minds of patients. It helps to face problems and provides higher inner peace, hope and optimism^29^.

When comparing the quality of life of adolescents with and without cleft lip and palate, we observed that the overall quality of life domain was significantly higher in adolescents with cleft lip and palate. The improvement of the Quality of life is associated with the perception of the meaning of life, the reassessment of opinions about illness and death, the discovery of new relations with God and the support of social relations. Spirituality and social relations may help in coping with pathology^7^. It should be emphasized that one of the pillars of the process of rehabilitation of patients with cleft lip and palate aims to improve the quality of life^1^ ^-^ ^2^.

The score in the Environment domain was also significantly higher in the case group, indicating that adolescents with cleft lip and palate presented better perception of the environment in which they live and of its structural aspects of life.

The literature states that people with high spirituality and religiosity have a positive correlation with the environment, psychological, social relationships and overall quality of life domains^26^ ^-^ ^28^.

We believe that after the diagnosis of a disease and the experience of a health problem, people seek a new meaning to life, becoming less likely to be bothered by small situations of everyday life that they used to allow affect their quality of life^26^ ^,^ ^29^.

In the correlation between the domains of quality of life and religiosity and spirituality in adolescents with cleft lip and palate, only the correlation between spirituality and global quality of life was identified. Contrary to our findings, other studies have correlated spirituality and/or religiosity with a better perception of quality of life in several dimensions^7^ ^-^ ^8^. However, higher levels of spirituality were associated with general well-being in another research^30^.

The results of the present study reinforce the hypothesis that spirituality encompasses a broad and dynamic concept, capable of influencing meanings or perceptions^31^. Another hypothesis that could explain this result is that adolescents are influenced by the spiritual and religious contexts of their parents, and sometimes end up replicating these values, although they have not incorporated them. Thus, their benefits are partial^31^ ^-^ ^32^.

Finally, the limitations of this investigation refer to the monocentric nature and the cross-sectional design which do not allow the establishment of causal relations or the generalization of the results. Thus, multicentric and longitudinal studies are encouraged. We must also consider the fact that the instruments used to collect data in this study have not yet been validated for the Brazilian culture and for the population studied.

However, the benefits of this research are evident, and include a detailed investigation of religiosity and spirituality in adolescents with and without cleft lip and palate and their correlation with quality of life. Although no correlations were detected between the majority of variables and quality of life, the study made it possible to identify that the perception of global quality of life was influenced by spirituality.

## Conclusion

Contrary to our hypothesis, there was a correlation only between spirituality and global perception of quality of life among adolescents with cleft lip and palate, indicating the absence of a relationship between religiosity and spirituality with quality of life in the majority of the aspects evaluated.
